# Multi-level perception fusion dehazing network

**DOI:** 10.1371/journal.pone.0285137

**Published:** 2023-10-02

**Authors:** Xiaohua Wu, Zenglu Li, Xiaoyu Guo, Songyang Xiang, Yao Zhang

**Affiliations:** 1 School of Art and Design, Sanming University, Sanming, Fujian, China; 2 Network Center (Information Construction Office), Sanming University, Sanming, Fujian, China; 3 School of Resources and Chemical Engineering, Sanming University, Sanming, Fujian, China; 4 Geography and Ecological Environment Research Center, Fuzhou University, Fuzhou, Fujian, China; 5 College of Education Sciences, Northwest Normal University, Lanzhou, Gansu, China; University of Engineering & Technology, Taxila, PAKISTAN

## Abstract

Image dehazing models are critical in improving the recognition and classification capabilities of image-related artificial intelligence systems. However, existing methods often ignore the limitations of receptive field size during feature extraction and the loss of important information during network sampling, resulting in incomplete or structurally flawed dehazing outcomes. To address these challenges, we propose a multi-level perception fusion dehazing network (MPFDN) that effectively integrates feature information across different scales, expands the perceptual field of the network, and fully extracts the spatial background information of the image. Moreover, we employ an error feedback mechanism and a feature compensator to address the loss of features during the image dehazing process. Finally, we subtract the original hazy image from the generated residual image to obtain a high-quality dehazed image. Based on extensive experimentation, our proposed method has demonstrated outstanding performance not only on synthesizing dehazing datasets, but also on non-homogeneous haze datasets.

## Introduction

Visible imaging devices are the primary means of capturing visual information, but their effectiveness is often hindered by adverse weather conditionsVisible imaging devices are the primary means of capturing visual information, but their effectiveness is often hindered by adverse weather conditions [1, 2]. This is especially problematic for computer vision applications that need to operate in various outdoor environments, as they may encounter weather-related challenges such as haze, which can interfere with their proper functioning. Given the high cost of hardware upgrades to address this issue, many researchers are exploring the development of advanced processing algorithms as a more cost-effective and scalable solution to this challenge [3–5].

Traditional dehazing methods [6–8] enhance image contrast or correct its color from the spatial domain or frequency domain or estimate unknown parameters in the model by combining the atmospheric scattering model with some prior knowledge, such as the color-line prior [9] and dark channel prior [10]. However, from the point of view of mathematical formulas, solving any unknown quantity in the atmospheric scattering model will be a very morbid process, that is easily affected by complex, diverse and changing scenes. Therefore, the restored image will be overenhanced or stylized, and the effect is not ideal.

Convolutional neural networks have emerged as a powerful tool for addressing image dehazing challenges by estimating transmission or directly predicting clear images [11–13]. While effective and superior to traditional algorithms, these deep learning methods require large amounts of hazy and clear image pairs for training, which can be impractical to obtain in real-world settings. The current mainstream image dehazing methods all use synthetic datasets, but these datasets only describe the form of uniform haze and the situation under normal lighting conditions, which differs from real-world scenes. Additionally, existing data-driven methods often overlook the limitations of traditional convolutional kernels, which can only extract features at fixed scales and may result in degraded image quality. To address these challenges, a multi-stream network architecture may offer a feasible solution by accounting for uneven mist distribution and different degradation levels across varying scene depths. However, simple parallel processing and mutual fusion may not be sufficient to fully leverage feature information extracted at different scales and prevent information loss during the sampling process [14–16]. Thus, a more sophisticated approach is needed to effectively restore image details and avoid common issues such as color distortion, texture loss, and halo artifacts.

The motivation behind our proposed method is to improve on previous limitations of image dehazing methods, specifically the fixed receptive field size in feature extraction that results in the loss of important information. To tackle this, we introduce MPFDN—a novel approach to image dehazing. Our approach utilizes a multi-level perception fusion module that allows for adaptive haze feature extraction from various receptive fields of different scales. This overcomes the limitations of the fixed receptive field size, and by fusing the feature information extracted at different scales, we share and retain effective features through an error feedback mechanism that mitigates the loss of significant information during the sampling process. By subtracting the residual mapping from the hazy image, we obtain a clear image. To enhance the accuracy of our model, we design a feature compensator that uses the covariance map generated by our embedding process to approximate the optimal residual image. Overall, our proposed MPFDN approach offers contributions to the field of image dehazing, the main contributions are as follows:

- We propose MPFDN, a method for generating a residual map from a hazy image to obtain a clean image.
- We introduce a multi-level perception fusion module for adaptive haze feature extraction from multiple receptive fields of different scales to overcome the limitations of the receptive field.
- We design a feature compensator to solve the model error and approximate the optimal residual image using the covariance map generated from embedding.

## Related work

Image dehazing is a challenging and ill-posed problem in computer vision. To tackle this problem, existing research work [4, 17–20] in this field can be broadly divided into two categories: traditional dehazing methods and data-driven dehazing methods.

### Traditional dehazing methods

The purpose of image enhancement-based dehazed algorithms is to eliminate as much noise as possible and improve the contrast of the image to achieve a hazy-free restoration. Typical algorithms are histogram equalization, retinex algorithm [21–23] and homomorphic filtering. However, in the presence of haze, this method can lead to excessive local enhancement of the image and even severe distortion and artifacts.

The image-restoration-based dehazing algorithm performs mathematical modeling, such as a physical model of atmospheric scattering, based on the causes of image degradation and then estimates the unknown quantities in the model to recover haze-free images. Narasimhan et al. [24] synthesized and established the depth model of the scene and then recovered the haze-free image based on this depth model and achieved a good dehazing effect. Based on a priori knowledge, the contrast of the haze-free image is higher than that of the hazy image. Tan et al. [25] performed image dehazing by solving for the maximum value of the local contrast of the hazy image. This method usually results in oversaturation of the recovered image and tends to produce halos. The assumption is that the reflectance of a small local area is fixed and uncorrelated with the propagation rate. Fattal et al. [26] solved the transmittance map by estimating the reflectance to achieve image recovery. This algorithm performs the dehazing process based on the statistical values of the image color information and once there is insufficient color information in the image, the statistical values are not general, and the dehazing effect is likely to be poor. He et al. [10] proposed the dark channel a priori theory, which can achieve simple and fast dehazing. Tarel et al. [27] estimated the transmittance by median filtering and obtained the dehazed image by atmospheric scattering model. Zhu et al. [28] proposed an a priori theory of color decay to learn linear functions to predict the depth of hazy images. Berman et al. [29] proposed a non-local a priori dehazing method. Zhang et al. [30] proposed a dehazing algorithm combining dual-region filtering and image fusion, which can effectively reduce the halo effect. Wang et al. [31] improved the accuracy of transmittance estimation with the help of superpixel segmentation. Dhara et al. [32] proposed an effective dehazing technique is proposed using weighted least squares filtering on dark channel prior and color correction that involves automatic detection of color cast images.

### Data-driven dehazing methods

With the rapid development of deep learning in various fields [4, 33–35], a large number of deep learning-based image processing algorithms [36, 37] have been proposed, and substantial progress has been made.

At first, researchers combined traditional methods with data-driven methods for image processing [38]. Cai et al. [11] and Ren et al. [12] constructed CNNs to estimate the transmittance and substitute them into the imaging model to obtain the recovered images. However, they set the atmospheric light as a global constant, and the dehazed images are prone to artifacts and color distortion. Zhang et al. [13] constructed a densely connected pyramidal network to estimate both transmittance and atmospheric light. Li et al. [39] combined transmittance and atmospheric light into one variable K and constructed a lightweight network AOD-Net to estimate K. All the above methods rely on physical atmospheric models, which limits the learning ability of the network to some extent.

To solve the problem of excessive dependence on models, an end-to-end network algorithm structure was [40] proposed that depends on a large amount of data. Later, it was widely used in image dehazing. Chen et al. [41] proposed an end-to-end gated contextual aggregation network based on the introduction of smoothly expanding convolution, which can fuse features at different levels. Liu et al. [42] constructed a multiscale grid network based on the attention mechanism, which used a parallel multiscale mechanism to solve the error transfer problem that is common in serial multiscale networks, but the network ignored the connection between non-adjacent scales. Yang et al. [43] reconstructed haze-free images by aggregating multiscale feature maps, but the network structure is simple and requires multiple downsamplings of the original image, which may cause loss of image details. Zhang et al. [44] proposed a multilevel fusion module to utilize both low-level and high-level features. The low-level features help to recover finer details, and the high-level features discover abstract semantics. Zhang et al. [45] proposed a network combining multiscale hierarchical feature fusion and mixed convolution attention to progressively and adaptively enhance the dehazing performance. Li et al. [46] proposed a dehazing framework based on conditional normalizing flow, which involves learning the conditional distribution of haze-free images to generate multiple dehazed results. Li et al. [47] proposed a self-supervised dehazing framework that does not rely on paired datasets, based on the prior that the difference between the brightness and saturation in the haze-free area of an image is close to zero and using the atmospheric scattering model. This multiscale feature fusion method considers the extraction of finer features but neglects the sharing of feature information between different scales and the refinement of feature details during the sampling process.

## Method

**Fig 1:**
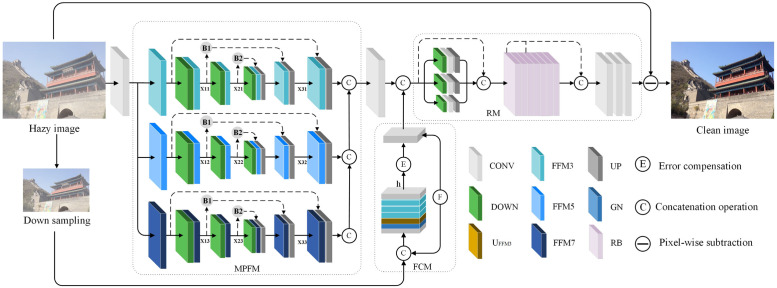
Overview of the proposed method. First, hazy image is input to MPFDN, and result of loss compensation module is combined and input to the refinement module for better adjustment of clustered feature map. Finally, hazy image and residual map are subtracted to output a clear image. where $\overset{x}{\to }$ represents information flow with index *x*. RB stands for resblock abbreviation.

In this section, we introduce a novel method called MPFDN, which is illustrated in Fig 1. The proposed MPFDN effectively addresses the limited perceptual field issue with its multi-level perception fusion mechanism. Furthermore, an error feedback mechanism is incorporated to better integrate contextual information and compensate for the loss of some details during the sampling process. Another contribution of MPFDN is the design of a feature compensator that employs feedback to adjust the model error. Lastly, detail optimization is employed to fine-tune the feature maps of the clusters, thereby achieving a clear output image.

### Feature fusion module

**Fig 2:**
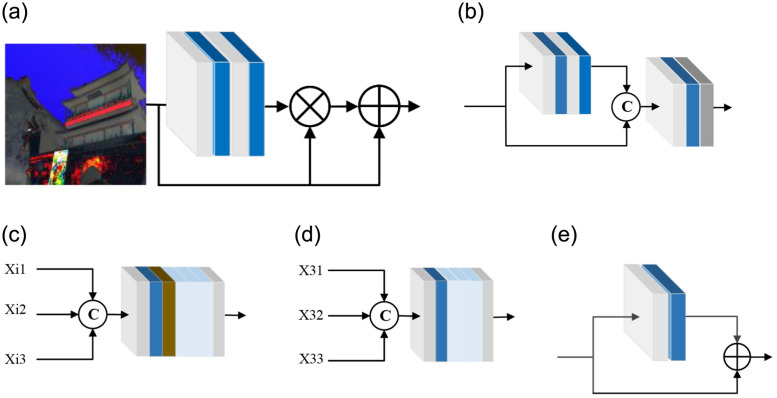
Left to right: Introductions of feature transformation module(FTM), feature fusion module (FFM), encoder-decoder branch, fusion module, RB.

For better feature fusion, we propose a feature fusion module (FFM), which uses group normalization [48] and an *SE* block [49]. The *SE* block provides proportional weighting factors. The more contextual information, the more feature channels, as shown in Fig 2. The gray block represents 3 × 3 convolution block, the blue block represents group normalization and the dark gray represents the *SE* block. The specific operation of *SE* block is as Eq (1):

$$\begin{array}{c} SE(x)=Sigmoid(fnn(ReLU(fnn(G(x)))))⊗x, \end{array}$$ (1)

where *G* represents the global average pooling, *fnn* represents the fully connected layer and ⊗ is pixel-wise multiplication.

### Multi-level perception fusion module

The proposed network is built on an encoder-decoder basis, an architecture widely used in image dehazing. The encoder-decoder module FFM generates a large receiver domain where contextual information can be obtained. Since haze usually has different shapes, densities and scales, some useful information may be lost by utilizing only singlestream networks [50–52]. Multi-scale convolutional kernel has been successfully applied in many fields [53, 54], which gives us inspiration. Therefore, we propose a multi-level perception fusion module (MPFM) to extract multi-scale focused features by setting the kernel sizes of the FFM belonging to their respective streams to 3, 5 and 7, respectively, to obtain more details, as shown in the *MPFM* section of Fig 1, the module architecture can be described as Eq (2):

$$\begin{array}{c} MPFM\left(x\right)=\left[U_{3\times 3},U_{5\times 5},U_{7\times 7}\right], \end{array}$$ (2)

where *U* denotes the *U*_*FFM*_ module with kernel size i, [] for serial operation.

### Error feedback mechanism

For the error feedback mechanism, an additional feature transformation module (FTM) and a concise encoder-decoder branch are introduced to generate high quality residual images and to obtain more information about the images. This section focuses on the adaptive extraction of the true value of the residual image by the feature transformation module (FTM) and the design of a simple encoder-decoder branch to learn the fusion of information from different streams in the MPFM module to obtain the residual image obtained in the model, respectively. The result generated by the feature transformation module is used as the target of the encoder-decoder branch learning and the loss between the two, as error feedback, so that the residual map generated by the model can be correctly guided.

Where the Feature Transformation Module (FTM), as shown in Fig 2, where ⊗ is pixel-wise multiplication, ⊕ is pixel-wise addition. The gray block represents 3 × 3 convolution block, the dark blue block represents group normalization block and the light blue block represents the FFM module.

In addition, the input of the encoder-decoder branch is the information obtained by fusing the information of different streams in the MPFM module. Finally, it is worth noting that the learned transform residual mapping is copied three times and embedded into three streams with jump connections, as shown in the MPFM module in Fig 1, in order to facilitate all the information interactions extracted between different streams, solving the previous simple multi-stream module, without taking into account the exchange of information between different streams and losing important details. where the encoder-decoder branch module architecture can be described as Eqs (3) and (4):

$$\begin{array}{c} B\left(x\right)=FFMB(U_{FFM}\left(GN\left(Conv\left(x\right)\right)\right)), \end{array}$$ (3)

$$\begin{array}{c} FFMB(X)=Conv(FFM(FFM(FFM(x)))), \end{array}$$ (4)

where *U*_*FFM*_ represents a simple encoder-decoder branch containing the *FFM* blocks with a convolution kernel size of 3 × 3, *GN* represents group normalization. Next, for the two constructed concise branches containing the *FFM* block learn the residual mapping and error mapping to obtain a better feature representation.

### Feature compensation mechanism

Generally, because of the complexity of the error distribution, the variable errors caused by uncertainty in the training process are difficult to remap by CNN. Therefore, we propose a feature compensation mechanism (FCM), which ensures that the features extracted from different scales are normalized to have similar statistics before being combined. As shown in Fig 1, FCM mainly includes the Fusion module(FM) and embedded residual mapping. This embedded module makes up for the uncertain loss in the previous MPFM module training.

For FM, feature extraction processes the upsampling three times (see Fig 2) with a concise branch, which integrates deep features in multi-stream networks, the function can be formulated as Eq (5):

$$\begin{array}{c} FM=Conv(GN\left(FFMB\left(x_{31}⊕x_{32}⊕x_{33}\right)\right)). \end{array}$$ (5)

Subsequently, as shown in Fig 1, the FCM module fuses the original hazy image to a half-scale size after feature extraction for the embedded residual mapping to obtain the absolute error mapping map. The absolute value of error reciprocal can reduce the complexity of error reciprocal distribution. Our function can be formulated as (6) and (7):

$$\begin{array}{c} E=FM-err(1-2FM), \end{array}$$ (6)

$$\begin{array}{c} err=\frac{\theta }{h}-\theta , \end{array}$$ (7)

where *E* denotes the embedded residual mapping, *h* is the middle features of feature compensation mechanism (FCM), as shown in Fig 1. *θ* denotes the queueing parameter (set to 0.05 in the experiment). The middle features *h* is multipled by *θ* to denote the absolute error map *err*.

### Refine module

The main goal of the refinement module is to enhance the quality of the clustered features by using spatial pyramid pooling [55]. This is achieved by extracting multi-scale features with different scale factors, such as 4, 8, 16 and 32. The module employs point-by-point convolution and an interpolation operation to restore the original size of the feature maps. Furthermore, the module is designed to reduce the dimensionality of the feature maps and to enhance their accuracy. The top-down structure can be formulated as Eq (8):

$$\begin{array}{c} D(x)=[M(x),x], \end{array}$$ (8)

where the specific operational details of *M*(*x*) is formulated as Eq (9):

$$\begin{array}{c} M\left(x\right)=\left(c\left(x{↑}_{4}\right)\right){↓}_{4},\left(c\left(x{↑}_{8}\right)\right){↓}_{8},\left(c\left(x{↑}_{16}\right)\right){↓}_{16},\left(c\left(x{↑}_{32}\right)\right){↓}_{32}, \end{array}$$ (9)

where *c* denotes the convolution abbreviation, ↑ is upsampling and ↓ is mean-pooling.

After using the spatial pyramid, we design a resblock group and perform group normalization [48]. residual block(RB) is designed as shown in Fig 2, where ⊕ is the pixel addition and the residual block containing the normalization. Because the performance of selecting group normalization is better than that of instance normalization and batch normalization when dealing with small batches, this experiment chooses group normalization.

### Loss function

Our goal is to make the hazy image as close as possible to the Ground truth image after being processed by MPFDN. Therefore, we adopt a hybrid loss function consisting of Structural Similarity Index (SSIM) loss and L1-norm loss to train MPFDN. Specifically, the SSIM loss is applied to evaluate the structural similarity, which can better preserve high-frequency structure information. L1 norm loss to constrain the differences between the color and luminance characteristics of the feature maps. These two loss functions can be formulated as Eqs (10) and (11):

$$\begin{array}{c} L_{1_{j}}=\frac{1}{N}\sum_{i=1}^{N}∥B_{j}-\text{GR}∥, \end{array}$$ (10)

$$\begin{array}{c} L_{s}=1-\text{SSIM}(R,\text{GT}), \end{array}$$ (11)

where $L_{1_{j}}$ and *L*_*s*_ are defined as the L1 norm and SSIM loss functions, respectively. GR represents the true residual diagram output by the FTM module, *B*_*j*_ represents B1, B2, R is the dehazed image and GT is the ground truth image, in the MPFM module in Fig 1. By combining the weighted sum of SSIM and L1 norm loss, our final hybrid loss function can be expressed as Eq (12):

$$\begin{array}{c} L_{total}=L_{s}+L_{1_{1}}+\lambda L_{1_{2}}, \end{array}$$ (12)

## Experiments and analysis of results

Our experimental study aims to address the following fundamental questions: 1) How effective is the proposed MPFDN framework? 2) What is the individual contribution of each component of MPFDN to its overall performance? To achieve this objective, we construct MPFDN with fundamental backbone networks to eliminate the performance boost gained from sophisticated network architectures. Furthermore, we benchmark the performance of MPFDN against several state-of-the-art methods across diverse datasets.

### Experimental setup

#### Datasets

To fully evaluate the performance of MDFDN, we conduct tests on several baseline datasets(synthesizing dehazing dataset: RESIDE [58], non-homogeneous haze datasets: I-HAZE [59], O-HAZE [59], NH-HAZE [60] and the NTIRE2021). Among them, the RESIDE dataset includes multiple sub-datasets. We mainly use ITS and OTS for training and SOTS for testing. The I-HAZE, O-HAZE, NH-HAZE and NTIRE2021 datasets are mainly used to evaluate the dehazing performance of the model in heterogeneous haze scenes. To ensure objectivity and impartiality, we conducted separate training and testing on the datasets used to compare the proposed method with existing ones.

#### Training details

The operating system used for the experiments is Ubuntu 20.04.3, the GPU configuration is NVIDIA RTX 3090 24GB × 2. The deep learning architecture used for training is PyTorch, the epochs are 20 in the RESIDE dataset, the epochs are 200 in the real scene dataset and the batch size is 3. The models are optimized using the AdamW optimizer with an initial learning rate of 0.0003, and the learning rate is adjusted accordingly during the training process.

#### Quality measures

In order to evaluate the dehazing performance of the proposed network more objectively, PSNR (Peak Signal to Noise Ratio), SSIM (Structural Similarity), MS-SSIM (Multi-Scale Structure Similarity) and RMSE (Root Mean Square Error) metrics in this paper. PSNR measures the ratio between the maximum possible power of a signal and the power of corrupting noise that affects the fidelity of its representation. SSIM and MS-SSIM measure the similarity between two images based on their luminance, contrast, and structural information. RMSE measures the average magnitude of the differences between predicted and actual values in a set of data. Through these index tests, it can be proved that our proposed method has a better dehazing effect and retains the original image features.

### Performance evaluation

Table 1 shows the quantitative evaluation results of current popular methods and MPFDN. Each row in Table 1 indicates the average of the test results on different datasets and each column indicates the average of the test results using the same method on different datasets. It is worth noting that the red font in Table 1 represents the best and the blue font represents the second best. Combining the performance of SSIM under all datasets, our proposed method is about 10% higher than the current best solution (GDN), while combining the performance of PSNR, MS-SSIM and RMSE under all datasets, our proposed method is about 21%, 9% and 37% higher than the current best solution (MSBDN) respectively. It can be seen that our MPFDN algorithm is overwhelmingly better than other algorithms in PSNR, SSIM, RMSE and MS-SSIM metrics.

**Table 1:** Quantitative evaluation on benchmark dehazing datasets.

Baseline	Metric	DCP [10]	NLD [29]	GDN [42]	MSBDN [56]	DeFlow [46]	YOLY [47]	RDN [57]	Ours
SOTS	SSIM↑	0.844	0.808	**0.969**	0.927	0.722	0.801	0.877	0.957
	PSNR↑	18.50	17.52	31.55	29.53	23.72	19.65	20.60	**31.64**
	MS-SSIM↑	0.923	0.908	0.988	0.983	0.888	0.942	0.951	**0.989**
	RMSE↓	32.67	35.57	7.30	9.87	18.06	35.22	25.22	**6.96**
I-HAZE	SSIM↑	0.651	0.721	0.685	0.739	0.602	0.739	0.753	**0.839**
	PSNR↑	12.94	14.21	13.05	16.29	13.87	15.10	15.91	**23.15**
	MS-SSIM↑	0.739	0.808	0.796	0.797	0.809	0.789	0.856	**0.936**
	RMSE↓	58.13	51.35	60.98	42.17	53.64	46.91	43.43	**20.74**
O-HAZE	SSIM↑	0.575	0.636	0.801	0.639	0.645	0.561	0.772	**0.835**
	PSNR↑	16.17	15.44	19.25	18.38	16.89	17.15	17.06	**24.75**
	MS-SSIM↑	0.831	0.880	0.898	0.792	0.708	0.852	0.848	**0.943**
	RMSE↓	38.19	36.35	31.35	33.87	43.59	36.28	36.03	**18.54**
NH-HAZE	SSIM↑	0.53	0.575	0.647	0.658	0.623	0.359	0.561	**0.743**
	PSNR↑	13.01	12.71	15.43	17.46	17.09	11.73	13.12	**20.15**
	MS-SSIM↑	0.658	0.712	0.732	0.784	0.778	0.564	0.661	**0.839**
	RMSE↓	58.55	60.05	46.82	36.56	37.65	66.94	57.71	**30.61**
NTIRE2021	SSIM↑	0.64	0.635	0.695	0.781	0.757	0.412	0.657	**0.828**
	PSNR↑	12.16	11.08	14.56	17.81	17.37	10.82	12.23	**20.84**
	MS-SSIM↑	0.783	0.784	0.786	0.862	0.878	0.658	0.773	**0.908**
	RMSE↓	63.34	72.30	50.55	35.68	34.99	74.49	63.83	**27.88**
Efficiency	Flops(GFlops)↓	-	-	**21.42**	41.52	-	-	75.41	70.38
	Params(MB)↓	-	-	**0.96**	31.35	151.00	32.01	65.80	5.80

**Fig 3:**
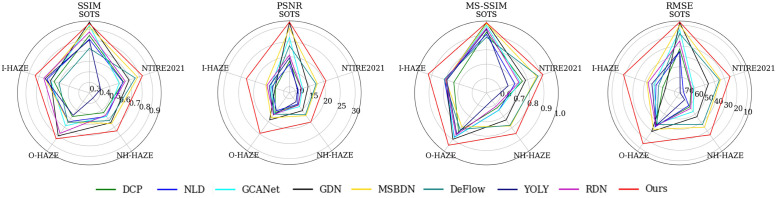
Comparison of different methods under each dataset for the same objective metrics.

**Fig 4:**
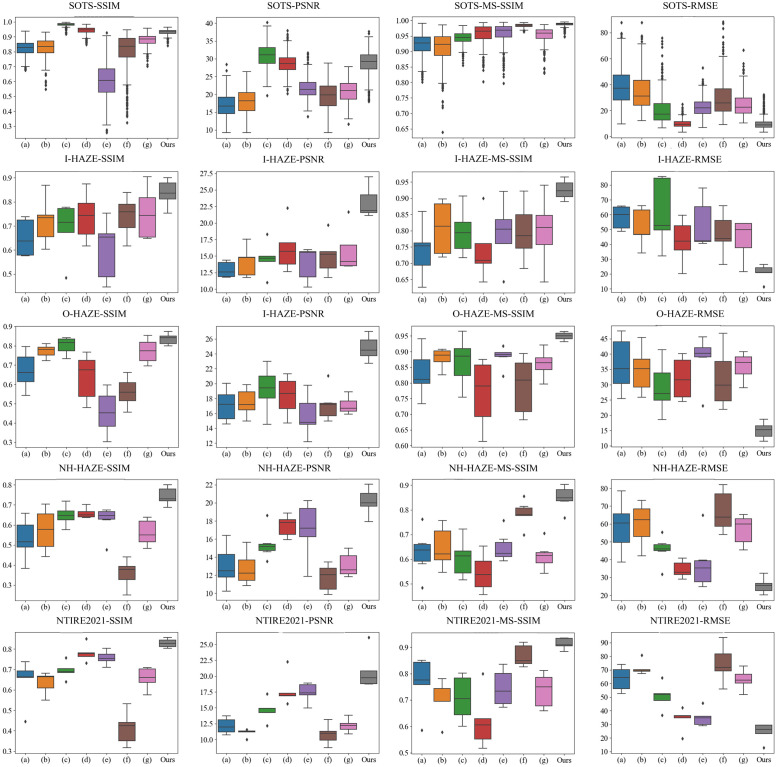
The data distribution of the quantitative evaluation results obtained by the popular quantitative evaluation methods in the data set is shown as a box plot. (a)-(h) represent DCP [10], NLD [29], GDN [42], MSBDN [56], DeFlow [46], YOLY [47] and RDN [57] respectively.

We utilize a radar chart to provide a visual comparison of different methods across each dataset, allowing for a more intuitive understanding of the data (as shown in Fig 3). Additionally, the box plot (as displayed in Fig 4) effectively conveys the distribution of data, and is employed to visualize the test results across all datasets. As illustrated in Fig 4, the performance of the various methods varies greatly as represented by their respective box plots. Compared to the current state-of-the-art method, MPFDN demonstrates more stable performance, as evidenced by the proximity between the upper and lower quartile lines.

**Fig 5:**
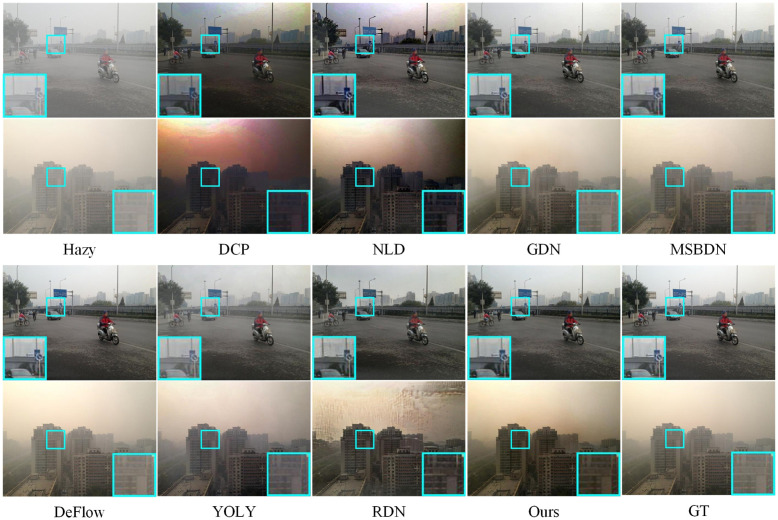
Visual results on the SOTS datasets (outdoor). Colored boxes are used to show the details of the images.

Fig 5 presents partial test results of current mainstream methods on the SOTS (outdoor) test set. The images processed by DCP exhibit significant color distortion and artifacts, especially when the lighting in the image is low. The images processed by NLP also show color distortion, but it is less severe than DCP. DCP and NLD both rely on prior assumptions to perform image dehazing, and their effectiveness is compromised when the input image does not meet these assumptions. GDN, MSBDN, DeFlow, and our proposed method all belong to supervised learning methods, so their performance on SOTS is closer to the ground truth (GT) images, with the haze mostly removed and the details preserved well. It is worth noting that when GT images have defects, our method performs better than other supervised learning methods, mainly reflected in less residual haze and clearer image details. YOLY belongs to self-supervised learning methods, which do not perform as well as supervised learning methods on large-scale datasets, mainly reflected in more residual haze and less clear details in the processed images. However, the color of the processed images by YOLY is more realistic compared to other supervised learning methods. RDN is a combination of supervised learning and DCP, and its performance is influenced by DCP. When the input image does not meet the prior assumptions of DCP, RDN will also fail.

We compare the proposed MPFDN with DCP [10], NLD [29], GDN [42], MSBDN [56], DeFlow [46], YOLY [47] and RDN [57] on SOTS, I-HAZE, O-HAZE, NH-HAZE and NTIRE2021.

**Fig 6:**
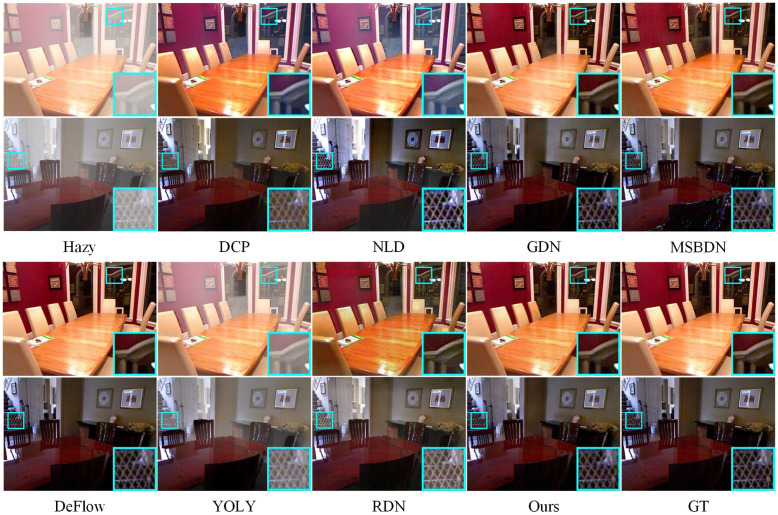
Visual results on the SOTS datasets (indoor). Colored boxes are used to show the details of the images.

Fig 6 presents partial test results of current mainstream methods on the SOTS (indoor) test set. The difference between SOTS (indoor) and SOTS (outdoor) is that the former uses depth information captured by equipment, while the latter obtains depth information through single-view depth estimation, making the hazy images in SOTS (indoor) more consistent with the atmospheric scattering model. As shown in Fig 6, methods based on prior assumptions (DCP and NLD) still exhibit certain color distortion and poor detail performance. GDN, MSBDN, DeFlow, and our proposed method are more color-consistent with GT images as a whole, but MSBDN’s processed images have artifact colors in dark areas. YOLY’s ability to handle heavily interfered images is weak, especially in deep scenes, where there is still a lot of haze residue. RDN’s processed images have the problem of low picture smoothness, and the image details deviate greatly from the GT images. Through comparisons on the SOTS test set, our proposed method has advantages in color, haze residue, and image details.

**Fig 7:**
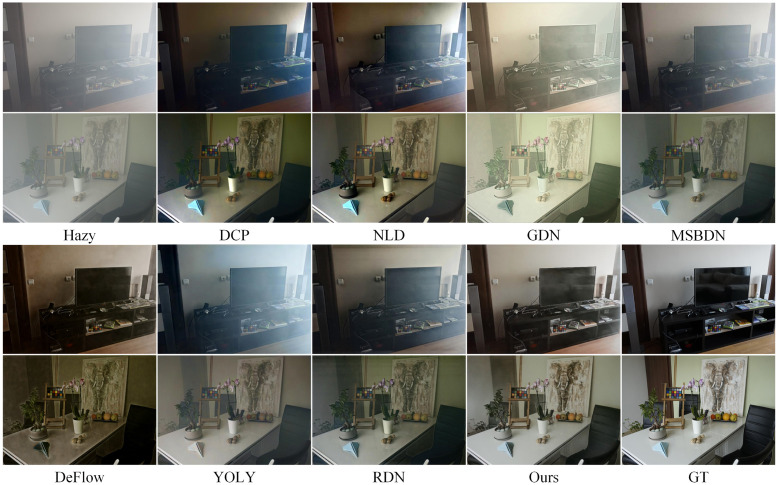
Visual results on the I-HAZE datasets.

**Fig 8:**
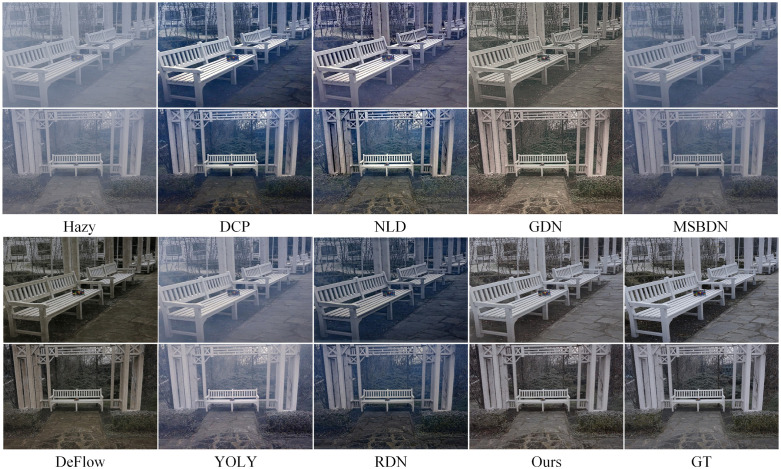
Visual results on the O-HAZE datasets.

**Fig 9:**
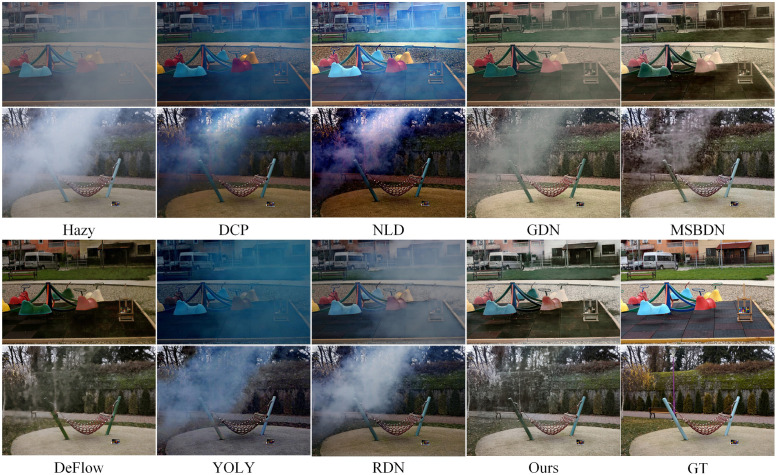
Visual results on the NH-HAZE datasets.

**Fig 10:**
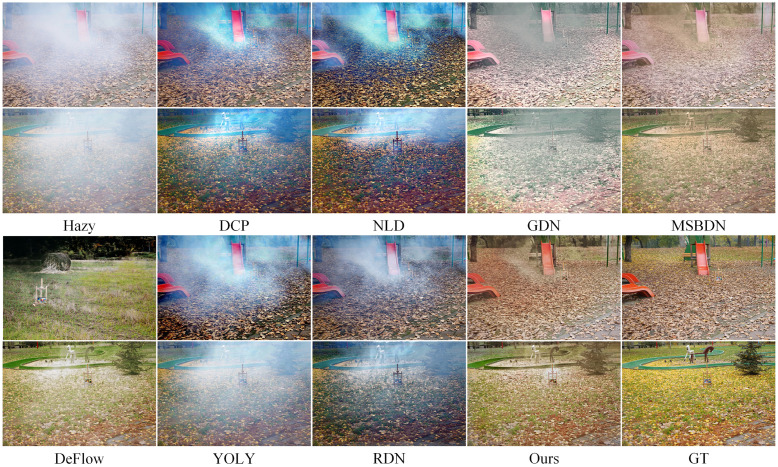
Visual results on the NTIRE2021 datasets.

Figs 7 to 10 show partial test results of different methods on a non-uniform haze dataset. The non-uniform haze dataset aims to simulate haze using smoke. In contrast to the I-HAZE and O-HAZE datasets, where the smoke is relatively uniform, the NH-HAZE and NTIRE2021 datasets focus on examining the processing effect of local haze. Unlike simulated datasets such as RESIDE, the non-uniform haze dataset uses smoke to occlude the scene. Smoke and water vapor in the haze have different physical properties, and the dataset has a smaller scale, making it more challenging than RESIDE. Especially in cases of local smoke such as NH-HAZE and NTIRE2021, smoke in the image is unrelated to depth of field, rendering methods based on atmospheric scattering models almost ineffective (as seen in DCP, NLD, YOLY, and RDN in Figs 9 and 10). However, methods based on supervised learning (such as GDN, MSBDN, DeFlow, and ours) learn the relationship between the haze image and the ground truth through training, resulting in less haze residue in the processed image. Nevertheless, there is still a certain degree of color distortion and detail loss, which is a common problem in current methods. In the relatively uniform smoke of I-HAZE and O-HAZE datasets, methods based on atmospheric scattering models have less of an impact but still have residual haze and under-saturation. Overall, our proposed method has more harmonious colors and less haze residue than other methods. It has advantages in both the overall visual impression and detail representation compared to other methods.

### Ablation study

**Fig 11:**
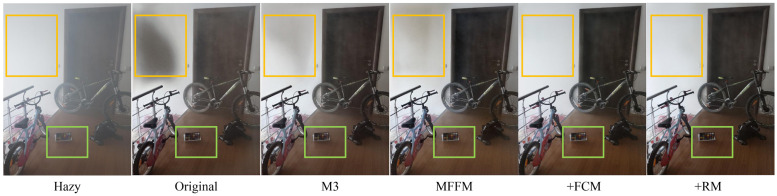
Experimental results of models with different configurations. The color boxes represent areas where there is a significant difference.

To further verify the effectiveness of each module of the algorithm in this paper, ablation studies were conducted on the I-HAZE dataset and two metrics, PSNR and SSIM, were used to measure the image quality. The experimental results are shown in Table 2, where original denotes the singlestream U-HRB module and no other modules are included. The original network itself makes the PSNR and SSIM reach 18.47 and 0.798, respectively. M3 indicates the conversion of the original single-stream mechanism into a multi-stream mechanism, as shown in Fig 1, and the MPFM part indicates the addition of the error feedback mechanism on top of M3 +FCM indicates the addition of FCM module on top of MPFM, and our indicates the addition of RM details on top of the above-module part. We combine the index results of the ablation experiments (as shown in Table 2) and the comparison charts of the ablation experiments (as shown in Fig 11) for analysis. It is not difficult to find that: (1) Original: We choose to simply use our proposed FFM module for single-stream network dehazing. From the results, there is a certain dehazing effect, but there are still problems of loss of details and large-area distortion. (2) M3: In response to the above problems, we propose a multi-stream network that aggregates feature information of different scales through the network to increase the network receptive field to fully extract image spatial context information. From the results, this module played a certain role and obtained more feature information, thus solving the problem of large-area distortion to a certain extent. (3) MPFM: We add the error feedback module to the original multi-stream network to better learn the details and avoid the loss of details caused by the downsampling process. From the results, we improved the detail. (4) +FCM: By merging the information of the upsampling process with the preprocessed original image, the image of the previous module is compensated in detail to obtain a clearer image. (5) +RM: Fine-tune the feature maps generated by the previous operations to obtain a more accurate dehazed image.

**Table 2:** Ablation study on I-HAZE dataset.

Original	✓	✓	✓	✓	✓
M3		✓	✓	✓	✓
MPFM			✓	✓	✓
+FCM				✓	✓
+RM					✓
SSIM	0.798	0.814	0.822	0.842	**0.856**
PSNR	18.47	19.57	20.57	21.06	**22.44**

## Conclusions

In this work, we propose an MPFDN that does not depend on a physical model to construct the mapping from hazy images to clear images in an end-to-end manner. First, after extracting features at different scales, the feature information should be shared in the sampling process to increase the network perception domain and fully extract the spatial context information of the image. In addition, an error feedback mechanism is used to predict the target image more accurately. Second, a feature compensation mechanism is proposed to compensate for details that may be lost during the training. Through extensive experiments, we have proven that MPFDN can better process all kinds of hazy images and has more advanced performance than existing methods. Although MPFDN exhibits superiority in comparison with non-homogeneous haze datasets, it still requires corresponding training sets for model training during testing. Therefore, the existence of a single weight cannot simultaneously achieve high performance across all datasets, which is a major issue in supervised image processing. Continual learning provides a new perspective to address this challenge, and we will focus on investigating its application in image dehazing through continual learning in the future.
